# Primary Mediastinal Seminoma: A Diagnostic and Therapeutic Challenge With an Optimistic Outcome

**DOI:** 10.1155/crom/3286934

**Published:** 2026-03-06

**Authors:** Stephanie dos Santos Bueno, Roberta Borin Previtale, Mike Rocha Alves, Klinger Soares Faico-Filho

**Affiliations:** ^1^ Department of Internal Medicine and Laboratory Medicine, Escola Paulista de Medicina, Universidade Federal de São Paulo, São Paulo, Brazil, unifesp.br

**Keywords:** CARE guidelines, case report, germ cell neoplasms, mediastinum, seminoma

## Abstract

**Introduction:**

Primary mediastinal seminoma is a rare germ cell tumor that predominantly affects young men. Clinical presentation is variable, and diagnosis relies on imaging studies, tumor markers, and histopathological confirmation. This case report describes the diagnostic and therapeutic approach in a patient with mediastinal seminoma, in accordance with CARE guidelines.

**Case Report:**

A 33‐year‐old man presented with hoarseness, progressive dyspnea, dysphagia, and cough. Diagnostic investigation revealed a large mass in the anterior and middle mediastinum, which was confirmed as seminoma by biopsy and immunohistochemical analysis. Treatment with cisplatin‐based chemotherapy (BEP regimen) resulted in a favorable response and significant tumor reduction.

**Conclusion:**

Early diagnosis and appropriate treatment of primary mediastinal seminoma are essential for achieving a favorable prognosis.

## 1. Introduction

Germ cell tumors (GCTs) originate from primordial germ cells and most commonly arise in the gonads (testes and ovaries). However, they may also occur in extragonadal locations, primarily along the midline of the body, including the mediastinum, retroperitoneum, sacrococcygeal region, and pineal gland [1, 2]. Among extragonadal GCTs, primary mediastinal seminoma is a rare entity, accounting for a small proportion of all GCTs and mediastinal neoplasms [2, 3].

This tumor predominantly affects young men, typically between the third and fourth decades of life [3, 4]. Clinical presentation is highly variable, ranging from incidental findings on imaging studies to symptoms related to compression of adjacent structures, such as chest pain, dyspnea, cough, superior vena cava syndrome, hoarseness, and dysphagia [3, 5].

Diagnosis of primary mediastinal seminoma requires a multimodal approach that includes imaging studies (chest radiography, computed tomography [CT], and magnetic resonance imaging), evaluation of serum tumor markers (alpha‐fetoprotein [AFP], beta‐human chorionic gonadotropin [*β*‐hCG], and lactate dehydrogenase [LDH]), and histopathological and immunohistochemical confirmation obtained through tumor biopsy [4, 6].

Seminomas are highly sensitive to cisplatin‐based chemotherapy and radiotherapy, which results in high cure rates and favorable overall outcomes, particularly when the disease is diagnosed at an early stage and in the absence of nonpulmonary visceral metastases [6, 7].

This case report aims to describe the clinical presentation, diagnostic workup, therapeutic management, and clinical evolution of a patient with primary mediastinal seminoma, following the CARE (CAse REport) guidelines to ensure accuracy, transparency, and clinical relevance [8].

## 2. Case Description

### 2.1. Inclusion Criteria

The present case was included based on the following criteria: (i) histopathological and immunohistochemical confirmation of primary mediastinal seminoma; (ii) absence of a gonadal primary tumor after comprehensive staging; (iii) clinically significant mediastinal mass associated with compressive symptoms; and (iv) availability of complete clinical, radiological, laboratory, and therapeutic data.

### 2.2. Patient Information

A 33‐year‐old man, born and residing in São Paulo, Brazil, with no history of smoking or alcohol consumption, presented with progressive respiratory and upper digestive symptoms. His medical history was notable for keratoconus and generalized anxiety disorder. Family history revealed prostate cancer in his grandfather and two paternal uncles, as well as keratoconus in two sisters. The patient denied fever, night sweats, or unintentional weight loss prior to symptom onset. A 10‐kg weight loss reported approximately 4 months before the onset of symptoms was attributed to marathon training and was not considered disease related, as constitutional symptoms are not commonly associated with mediastinal seminoma [9].

### 2.3. Clinical Findings

Symptoms began approximately 2 months before diagnosis with intense hoarseness, odynophagia, and dysphagia, with partial improvement following corticosteroid therapy. Over the following weeks, symptoms progressed to include dyspnea on moderate exertion and worsening dysphagia, accompanied by choking and coughing during solid food intake. On physical examination, the patient was in fair general condition, tachypneic (respiratory rate of 30 breaths per minute), with an oxygen saturation of 95%. Pulmonary auscultation revealed bilateral vesicular breath sounds, reduced at the left lung base. No palpable lymphadenopathy was detected, and the remainder of the physical examination was unremarkable.

### 2.4. Diagnostic Assessment

**FIGURE 1:**
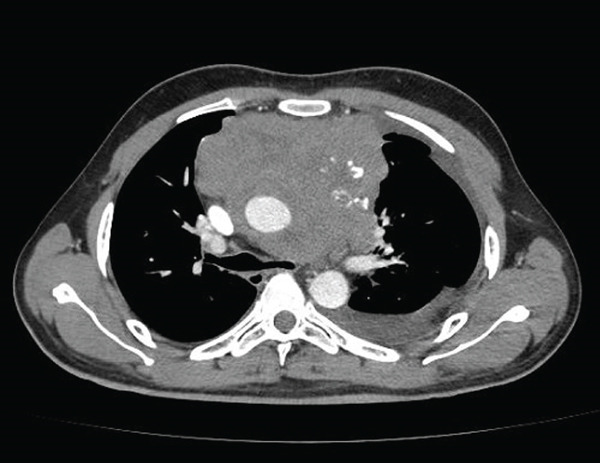
Heterogeneous mediastinal mass. Heterogeneous infiltrative mass in the anterior mediastinum, associated with superior vena cava narrowing and left pleural effusion.

**FIGURE 2:**
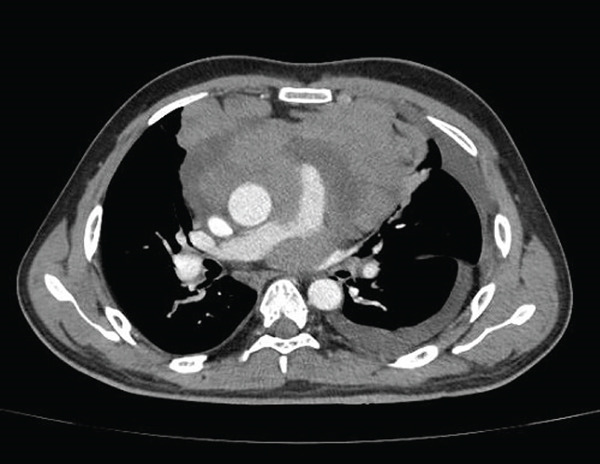
Pulmonary artery trunk narrowing. Large infiltrative mediastinal mass, compressing and narrowing the main pulmonary artery trunk and its branches.

**FIGURE 3:**
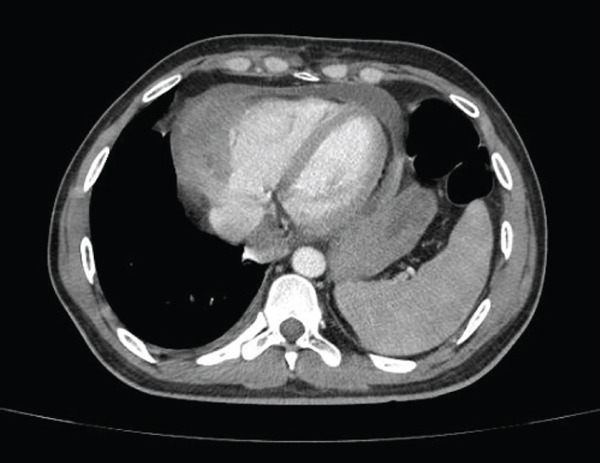
Pericardial effusion and diaphragmatic elevation. Moderate pericardial effusion with loss of the fat plane between the mass and the cardiac wall, and elevation of the left hemidiaphragm.

Videolaryngoscopy demonstrated medialization of the left vocal fold without edema or structural lesions. Contrast‐enhanced chest CT revealed a large infiltrative mass in the anterior and middle mediastinum, with irregular margins, heterogeneous contrast enhancement, and areas of calcification and necrosis (Figure 1). The mass measured 9.4 × 12.9 × 10.9 cm and involved the trachea and main bronchi, causing tracheal deviation and bronchial narrowing. Significant vascular involvement was observed, including narrowing of the superior vena cava and pulmonary artery trunk and branches (Figure 2), dilation of the jugular veins, and collateral circulation due to left brachiocephalic vein involvement. Loss of the fat plane between the mass and the cardiac wall suggested pericardial invasion, associated with moderate pericardial effusion (Figure 3). Additional findings included anterior diaphragmatic lymphadenopathy (up to 1.5 cm) and moderate left pleural effusion.

Laboratory evaluation revealed elevated levels of C‐reactive protein and LDH, along with minimally elevated *β*‐hCG levels (16.6 mIU/mL), while AFP levels were within the normal range (3.9 ng/mL), a laboratory profile consistent with seminoma. Ultrasound‐guided biopsy of the mediastinal mass demonstrated an undifferentiated high‐grade malignant neoplasm composed of monomorphic epithelioid cells arranged in a solid pattern, with areas of coagulative necrosis. Immunohistochemical analysis confirmed the diagnosis of a GCT of the seminoma type.

During hospitalization, the patient developed worsening pericardial effusion associated with cardiac dysfunction, requiring drainage. Biopsy findings confirmed the absence of pericardial metastasis.

For staging purposes, scrotal ultrasonography, abdominal and pelvic CT, and cranial magnetic resonance imaging were performed and showed no abnormalities. Cervical CT was also unremarkable. Based on these findings, the final diagnosis was primary mediastinal seminoma, classified as good prognosis according to the International Germ Cell Cancer Collaborative Group (IGCCCG).

### 2.5. Therapeutic Interventions

Following diagnosis, the oncology team initiated systemic chemotherapy. The selected regimen was BEP (bleomycin, etoposide, and cisplatin), with a planned total of three cycles. The first cycle was administered during hospitalization. The choice of therapy was supported by the well‐established sensitivity of seminomas to cisplatin‐based chemotherapy.

### 2.6. Follow‐Up and Outcomes

**FIGURE 4:**
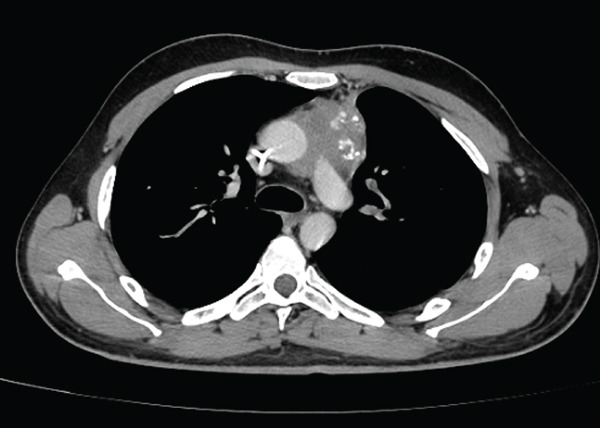
Residual mass after the third cycle. Reduction of the solid and heterogeneous anterior mediastinal mass, with calcifications, measuring 6.3 × 4.1 cm.

The patient demonstrated a favorable clinical and radiological response to chemotherapy. After the third BEP cycle, a marked reduction of the mediastinal mass was observed, measuring 6.3 × 4.1 cm on axial imaging (Figure 4). As planned, FDG PET‐CT performed 8 weeks after completion of chemotherapy demonstrated a residual mediastinal mass measuring 4.9 × 2.9 cm with moderate FDG uptake (SUV 4.3) and cervical lymph nodes suspicious for metastatic involvement. A subsequent follow‐up FDG PET‐CT showed further reduction of the residual mass (maximum diameter 3.9 cm) with decreased FDG uptake (SUV 3.5), along with metabolic regression of the previously suspicious cervical lymph nodes.

The patient remains under follow‐up with imaging and laboratory assessments to monitor treatment response, detect potential relapse, and manage late adverse effects (Table 1).

**TABLE 1:** Timeline of events.

Date/period	Event	Relevant details
Prediagnosis	Weight loss	10‐kg weight loss reported approximately 4 months before symptom onset.
Two months prior to diagnosis	Onset of symptoms	Hoarseness, odynophagia, and dysphagia, progressing to dyspnea, choking, and cough.
At diagnosis/hospitalization	Pericardial effusion	Increased pericardial effusion and cardiac dysfunction, requiring drainage.
After the third BEP cycle	Follow‐up CT	Residual mass measuring 6.3 × 4.1 cm.
Eight weeks after chemotherapy	Follow‐up PET‐CT	Residual mass measuring 4.9 × 2.9 cm (SUV 4.3). Cervical lymph nodes suspicious.
Four months after chemotherapy	Follow‐up PET‐CT	Residual mass measuring 3.9 cm in maximum diameter (SUV 3.5).

## 3. Discussion

Extragonadal GCTs are uncommon, with the anterior mediastinum representing the most frequent extragonadal site [2, 3]. Primary mediastinal seminoma predominantly affects young adult men and remains a rare subtype within this group [3, 4]. Several hypotheses have been proposed regarding tumor pathogenesis, including aberrant migration of primordial germ cells during embryogenesis, metastasis from a regressed gonadal primary tumor, or origin from thymic tissue [2, 5].

Clinical manifestations are often nonspecific and related to tumor size and compression of adjacent structures. Symptoms such as dyspnea, cough, dysphagia, and hoarseness, as observed in this case, are frequently reported [3, 5]. The absence of systemic symptoms, such as fever, night sweats, or unintentional weight loss, is consistent with existing literature [6].

Diagnosis relies on imaging findings, tumor marker profiles, and histopathological confirmation. Minimally elevated *β*‐hCG levels in the presence of normal AFP levels are characteristic of seminoma and help distinguish it from nonseminomatous GCTs [4, 6]. Histological confirmation remains the gold standard and is essential to differentiate seminoma from other anterior mediastinal tumors, including thymomas and lymphomas [4, 6].

Accurate staging is critical for treatment planning and prognostic assessment. Comprehensive imaging excluded gonadal and distant primary disease, confirming the mediastinal origin of the tumor.

Cisplatin‐based chemotherapy is the cornerstone of treatment for advanced seminoma. The BEP regimen is considered standard therapy, and the favorable response observed after the first cycle in this patient underscores its effectiveness [7]. Residual masses are common after treatment, and management depends on lesion size and metabolic activity on PET‐CT imaging, with surgical intervention reserved for selected cases [1, 5].

Overall prognosis for primary mediastinal seminoma is favorable, particularly in patients without nonpulmonary visceral metastases. Contemporary data report high cure rates with 5‐year survival exceeding 90% for seminomatous extragonadal GCTs, reinforcing the importance of early diagnosis and appropriate treatment [1, 5, 9].

## 4. Conclusion

Primary mediastinal seminoma is a rare but highly treatable malignancy with an overall favorable prognosis. This case highlights the importance of a thorough diagnostic evaluation and prompt initiation of cisplatin‐based chemotherapy. Adherence to CARE guidelines ensures accurate and transparent reporting, contributing to improved understanding and management of this uncommon condition.

## Funding

No funding was received for this manuscript.

## Consent

The patient provided informed consent for the publication of this case report. All information has been anonymized to protect his privacy and confidentiality, in compliance with ethical principles of research and scientific publication.

## Conflicts of Interest

The authors declare no conflicts of interest.

## Patient Perspective

Throughout the diagnostic and treatment process, the patient reported feelings of sadness but maintained an optimistic outlook regarding recovery. This positive attitude contributed to good adherence to treatment and coping with the challenges imposed by the disease.

## Data Availability

The data that support the findings of this study are available on request from the corresponding author. The data are not publicly available due to privacy or ethical restrictions.
